# Excision of the Superior Maxillary

**Published:** 1874-11

**Authors:** D. Keller

**Affiliations:** Paris, Ky.


### ARTICLE V.

### *Excision of the Superior Maxillary.*

BY D. KELLER, M. D., PARIS, KY.

In the month of March, 1869, assisted by Drs. L. D. Barnes, Washington Fithian and others, I removed the right superior maxillary bone of a negro woman aged fifty years.

This operation was rendered necessary by the disease of the antrum, which had existed for two years, and resisted all remedies applied for its removal, or mitigation of the intense suffering consequent upon it.

For more than a year, unless under the influence of morphia, there had been no rest, day or night. The operation having been determined upon, an incision was made, beginning at the inner canthus, extending to the angle of the mouth, and another from the alæ of the nose to the zygomatic fossæ, and thence in a perpendicular line to the temporal fossæ. The flaps now being dissected up and turned back, the anterior wall of the maxilla was brought into full view. With pliers, the division along the nasal line was effected, and with trephine, mallet and chisel, the subsequent steps for removal were completed.

In the removal of the orbital plate, the globe of the eye was carefully dissected up and raised, to prevent injury to that organ. The walls of the antrum were thickened to the extent of an inch, and required the use of the trephine in making an opening into the cavity, thereby lessening the difficulty in completing the removal, as it gave a good starting point for the saw or chisel. The orbital rim, being found in a healthy state, was not disturbed, while the floor of the orbit and posterior wall were necrosed, which were removed and actual cautery freely applied. After sponging out the wound thoroughly and all hæmorrhage had ceased, the flaps were brought down, properly adjusted and confined by sutures, a cold-water dressing was applied, and a full dose of morphia administered.

On inquiring next morning of my patient as to how she had spent the night, she replied that for the first time in a year she had a good night's rest

It is surprising how little deformity exists after such an operation, and how little the process of mastication is interfered with. With the exception of the facial scars, and slight caving in of the cheek, there was nothing left to show that such an operation had been performed. In six days the external wound had healed, and in a few weeks my patient had resumed her usual avocation, entirely relieved of all suffering, and remained well for two years, when she was attacked with dysentery and died, as I am informed.—*Medical Weekly.*

---

